# Supplementary material for: Evaluating Explainability: A Framework for Systematic Assessment of Explainable AI Features in Medical Imaging
Source: Bioengineering (Basel). 2026 Jan 16;13(1):111. doi: 10.3390/bioengineering13010111 (PMC12838233; doi:10.3390/bioengineering13010111)
Supplement: Supplementary file 1 [file bioengineering-13-00111-s001.zip › bioengineering-3990397-supplementary.pdf]

Supplementary Materials File S1: XAI Scorecard

The following ScoreCard is a mockup for the Ablation CAM explainability tool provided as an example of the template we propose.

SCORECARD

1. GENERAL INFORMATION

NAME

Ablation CAM

TYPE

Post-hoc

REACH

Local

DESCRIPTION

Ablation CAM systematically removes or perturbs parts of the model (such as feature maps or layers) to determine how each part contributes to the final prediction. By analyzing how the model's performance changes as parts are ablated, it generates a heatmap highlighting the most important regions or features influencing the model's decision.

REFERENCE

Ren, S., He, K., Girshick, R., & Sun, J. (2015). Faster R-CNN: Towards real-time object detection with region proposal networks. In Advances in Neural Information Processing Systems. (pp. 91-99).

SOFTWARE USED

grad-cam (version 1.5.2) with Torchvision

2. CONTEXT OF USE

INTENDED USER

Clinicians, AI Researchers

SPECIFIC TASK

Detecting lesions in digital mammography images

MODEL	DATASET
Faster R-CNN	M-SYNTH digital mammography

LIMITATIONS AND RECOMMENDATIONS

- Ablation CAM can become computationally expensive for large or deep models as it
  requires retraining or re-evaluating parts of the model during the ablation process.
- It may not perform well when applied to complex tasks requiring more nuanced or global explanations.
- Does not provide an explanation for absent lesion classifications
- Recommendations: Apply Ablation CAM to tasks where localized feature importance is key (e.g., lesion detection). Consider combining it with global methods like Grad-CAM to obtain a more holistic view of the model's decision process.

VALIDATION SETTING

- Ablation CAM was validated through quantitative metrics (e.g., SSIM, IoU) by comparing generated
  heatmaps with ground truth lesion locations in digital mammography images.
- A reader study with radiologists was conducted to assess clinical relevance and interpretability of the generated heatmaps.

3. QUANTITATIVE PERFORMANCE

Ablation CAM demonstrated strong performance across all five dimensions of explainability. It showed high consistency and fidelity, providing stable and faithful explanations. The plausibility and clinical relevance of its heatmaps were superior to those of Eigen CAM, and its results were aligned with the clinical task at hand. Additionally, the generated explanations were highly interpretable, making Ablation CAM a reliable and effective explainability method in medical imaging tasks such as lesion detection in digital mammography.

CONSISTENCY

- Structural Similarity Index Measure (SSIM): 0.87
- Intersection Over Union (IoU): 0.75 Mean Squared Error (MSE): 0.04

Results (Fig 2): Ablation CAM demonstrated high consistency across varying X-ray radiation doses and degrees of rotation. The performance was stable as indicated by metrics such as SSIM (Structural Similarity Index Measure), MSE (Mean Squared Error), and IoU (Intersection over Union).

Examples: The heatmaps generated by Ablation CAM remained relatively stable with minimal changes, even under different doses and rotations. This indi cates strong robustness and consistency in Ablation CAM's performance under different imaging conditions.

PLAUSIBILITY

- IoU with Ground Truth Bounding Box: 0.78
- Spearman Rank Correlation: 0.82

Results (Fig 3): Ablation CAM achieved higher plausibility compared to Eigen CAM, with a better alignment between the generated heatmaps and the ground truth lesion locations. This is reflected in the IoU values and the visual examples, where Ablation CAM more accurately highlighted the lesion locations.
Examples: High plausibility is shown where the heatmap aligns closely with the ground truth lesion (black square). Ablation CAM accurately centers the ROI (pink square) over the lesion in the high plausibility example, unlike Eigen CAM, which performed poorly in this regard.

FIDELITY

- Model Parameter Randomization Check: High Fidelity
- Change in IoU after Randomization: 0.68

Results (Fig 4): Ablation CAM consistently showed higher fidelity compared to Eigen CAM. The fidelity was evaluated through SSIM, IoU, and the Model Parameter Randomization Check. Ablation CAM demonstrated strong alignment with the model's internal reasoning, particularly under the randomization condition, where its heatmaps showed minimal changes.

Examples: In cases where model predictions did not change, Ablation CAM's heatmaps remained stable (small changes in heatmap), which reflects high fidelity. This contrasts with Eigen CAM, where larger changes in the heatmap were observed, indicating lower fidelity.

USEFULNESS

- Assessed through a reader study with radiologists: 4.5/5 (Likert Scale)
- Feedback: Heatmaps were useful for identifying key lesion areas

Qualitative Evaluation: Ablation CAM's heatmaps, especially in lesion detection, were highly useful for the clinical task. The method consistently highlighted the key regions (lesions) that are critical for diagnosis which are also easy to interpret. As seen in the examples, the highlighted areas directly contribute to the model's decisions and align with clinical expectations.

Quantitative Evaluation: Though not explicitly presented in the figures, Ablation CAM's higher plausibility and fidelity suggest that it would perform well in a clinical situation, as it provides explanations that clinicians can trust for making informed decisions.
